# Interactive, Multiscale Urban-Traffic Pattern Exploration Leveraging Massive GPS Trajectories

**DOI:** 10.3390/s20041084

**Published:** 2020-02-17

**Authors:** Qi Wang, Min Lu, Qingquan Li

**Affiliations:** 1 State Key Laboratory of Information Engineering in Surveying, Mapping and Remote Sensing, Wuhan University, Wuhan 430072, China; wangqi@whu.edu.cn; 2 Shenzhen Key Laboratory of Spatial Smart Sensing and Services, Shenzhen University, Shenzhen 518060, China; lumin.vis@gmail.com; 3 School of Architecture and Urban Planning, Shenzhen University, Shenzhen 518060, China

**Keywords:** traffic pattern, pattern recognition, visual analytics, traffic perception and exploration

## Abstract

Urban traffic pattern reflects how people move and how goods are transported, which is crucial for traffic management and urban planning. With the development of sensing techniques, accumulated sensor data are captured for monitoring vehicles, which also present the opportunities of big transportation data, especially for real-time interactive traffic pattern analysis. We propose a three-layer framework for the recognition and visualization of multiscale traffic patterns. The first layer computes the middle-tier synopses at fine spatial and temporal scales, which are indexed and stored in a geodatabase. The second layer uses synopses to efficiently extract multiscale traffic patterns. The third layer supports real-time interactive visual analytics for intuitive explorations by end users. An experiment in Shenzhen on taxi GPS trajectories that were collected over one month was conducted. Multiple traffic patterns are recognized and visualized in real-time. The results show the satisfactory performance of proposed framework in traffic analysis, which will facilitate traffic management and operation.

## 1. Introduction

Understanding urban traffic dynamics facilitates the lives of urban residents, the operations of transport managers, and the alleviation of air pollutions. With the development of information and communication technology (ICT), the Internet of things (IoTs), and the global positioning system (GPS), vehicle GPS trajectories have been widely collected in many cities [1,2,3]. As vehicles, especially taxis, move around the city, their GPS trajectories become important data sources for advanced traffic information systems and advanced traffic management systems [4,5]. Currently, raw GPS data are processed for real-time traffic monitoring or vehicle dispatching when they are streamed in. Sometimes, these data are stored for an additional 2 to 6 months or discarded directly. Due to the large volume of vehicle GPS data, it is difficult to process massive GPS data to perceive and explore the hidden traffic patterns in real-time. Consequently, it is essential to develop a new approach that has low storage and computing costs for exploring citywide traffic patterns.

Many studies have been conducted on the extraction of transport information. Several transport attributes have been recognized from various data sources, for example, road network [6], travel speed [7], travel volume [8], and traffic congestion [9]. A set of effective approaches has been developed for analyzing road traffic, which includes statistics-based methods [10,11,12,13] and clustering-based methods [14,15,16]. For example, Zou et al. [17] examined road traffic using long-term vehicle trajectories and identified the spatial dependency of the traffic state via spatial autocorrelation. Guo et al. [18] developed an effective computing system for investigating regular and abnormal traffic flow patterns at road intersections. These studies provide an effective analysis of traffic at various places. However, these methods are computation-intensive and, thus, have difficulty in real-time exploration analysis. In addition, due to the cascaded nature of urban traffic [17], congestion at an intersection may produce a vehicle queue at another road. The exploration of multiscale urban traffic patterns from one road to the city area is expected.

Meanwhile, due to its satisfactory performance on big data computing and prediction, machine learning methods have been applied in transport data analysis, such as tensor factorization [19], artificial neural networks [20], and graph neural networks (GNNs) [21]. Although complex traffic patterns have been extracted, a gap between the computed results and the associated human understanding remains. A more friendly interactive tool for the intuitive illustration of urban traffic is needed to enable users to understand citywide traffic efficiently.

We present a three-layer interactive framework for the exploration of multiscale traffic patterns leveraging massive GPS trajectory and the intuitive visualizations for effective perception. We propose a middle-tier data structure, namely, the synopses, which connects the data processing layer and the pattern recognition layer to increase the efficiency to enable real-time traffic exploration analysis. According to the end users’ interests, multiple traffic patterns are effectively recognized. In the visual analytic layer, a web-based system is designed to facilitate end users visualizing, perceiving, and exploring citywide traffic dynamics in real-time. Finally, using GPS data that were collected in Shenzhen City over one month, we conducted an experiment to evaluate the performance of the proposed framework.

The main contributions of this study are summarized as follows:

- A three-layer framework is proposed for real-time interactive traffic pattern exploration analysis on massive GPS trajectories.
- The synopses are proposed to constitute a middle-tier data structure to accelerate pattern recognition and support real-time exploration.
- A friendly interactive visual analytics system is developed for exploring urban road traffic dynamics intuitively.

The remainder of this article is organized as follows: Section 2 reviews the related literature. Section 3 introduces the study area and the dataset. In Section 4, we describe the proposed framework and method in detail. Section 5 reports a case study and presents several examples. In Section 6, we discuss the performance of the developed system. Finally, we present our conclusions in Section 7.

## 2. Related Work

### 2.1. Data-Driven Traffic Monitoring

Traffic monitoring has been a popular topic as it facilitates transport management and planning. Due to the advantage of passive sensing, increasingly many sensors are being used in the transport sector, such as probe vehicles, loops, and cameras [22,23,24,25]. These sensors produce abundant data that capture the urban traffic status, thereby enabling traffic monitoring. Consequently, data-driven methods are widely developed for monitoring traffic attributes, such as the speed, the flow, the occupancy, and the travel time [22,26,27,28]. These results are useful for forecasting traffic status, detecting traffic incidents, and planning transportation facilities [29,30,31,32]. 

Data-driven traffic monitoring studies contain two main parts: real-time traffic sensing and traffic pattern extraction. Real-time traffic sensing processes massive sensed data and produces traffic information for travelers and transport managers [30,33]. The main challenge is to infer the accurate traffic status despite the inhomogeneity and the sparseness of the sensed data. 

Traffic pattern extraction is another task of data-driven traffic monitoring. Three categories of methods have been developed for this task: probability-based methods, clustering methods, and neural network (NN) methods. The probability-based methods compute traffic properties such as speed and flow [10,13,34]. The distribution, randomness, and relationship among these properties are used to estimate parameters that describe the traffic patterns [11,12]. The clustering methods group road links according to their similarity in terms of traffic properties. The clustering methods have wide applications in traffic pattern extraction as they can consider various traffic features [14,15,16]. To obtain more accurate results, several NN methods are applied in traffic data analysis, such as local artificial neural networks [20], fuzzy neural networks [8], and sequence graph neural networks [21]. However, these methods focused mostly on fixed temporal and spatial scales; hence, they cannot explore dynamic traffic intuitively. 

Road traffic changes with space and time. Several studies represented road traffic as time-series sequences and examined the trends and recurrent patterns using the autoregressive integrated moving average model (ARIMA) and the Markov model [15,35,36,37]. Furthermore, spatial autocorrelations and cross-correlations are computed to generate relationships among road links [17,38,39]. Overall, these advanced studies have demonstrated the spatial-temporal variation of road traffic in the city. This study developed an efficient computing framework for supporting friendly and intuitive traffic exploration using massive GPS trajectories.

### 2.2. Traffic Visual Analytics

The revealed traffic patterns are typically complex; thus, they are difficult for users to understand. Recently, visual analytics of temporal, spatial, numerical, and categorical properties were presented for facilitating the interpretation of complex results by users [40]. Vehicle trajectories are represented as dots or polylines on a map [41,42]. Traffic visualization techniques are categorized into three main groups: direct depiction, derived data visualization, and extracted pattern visualizations [43]. For example, in combination with typical two-dimensional spatial maps, studies modeled the temporal dimension as the z-axis to depict the temporal properties [44,45]. The novel route-zooming technique [46] embeds the spatiotemporal information into a map, to avoid the visual clutter that arises in direct depiction, which enables us to display spatial information and attributes at the same time. For visualizing derived data, aggregation is a popular approach. Kernel density estimation is mostly used to derive the density of trajectories [47]. The hotspot map is another tool for indicating significant places behind massive traffic data [48]. By bundling massive vehicle trajectories, the changing flow visualization is proposed for mapping massive mobility flows [49]. Furthermore, studies have focused on the visualization of traffic patterns. For example, the propagation of a traffic jam is displayed on a spatial map to show how the traffic jam evolves from one road to another [50]. 

Visualization depicts the results using various visualization forms, whereas visual analytics focuses on methods “for an effective understanding, reasoning and decision making” [51]. Visual analytics is a useful and powerful analysis method for big data due to the advantage of combining human intelligence and machine computing. Various studies have proved the performance of visual analytics on transport problems. For example, a friendly trip visualization system, namely, TripVista [18], was developed for examining the movement behaviors at intersections, such as the object types, travel speeds, and travel directions. Traffic events are detected and displayed, such as traffic jam [50] and human gathering activities [52]. Visual analytics on the route diversity facilitate the determination of the usages of urban roads [53,54]. These studies of visual analytics enable the intuitive exploration of massive transport data. Following this direction, we put forward the mission designing visual analytics with knowledge of urban traffic to develop a data-driven interactive visual analysis system for exploring traffic pattern friendly and intuitively. 

## 3. Study Area and Datasets

We conducted this study in Shenzhen, China. Shenzhen is one of the most developed cities in southern China. It covers a total area of approximately 2000 km^2^. After fast development over the past forty years, Shenzhen has become a global city with 20 million residents and 3.4 million vehicles. Daily traffic has become a challenging issue for transport management and operations. We considered two categories of data: road network and vehicle GPS records. The road network of Shenzhen was provided by the Transport Commission of Shenzhen Municipality. The road network is represented by a graph of interconnected lines and points, in which lines denotes road links and points denotes the intersections of road links. The road links also contain several attributes, such as the name, the level, and the local administrative region. The road network is organized according to the topology. 

The vehicle GPS records were also provided by the Transport Commission of Shenzhen Municipality. This dataset contains the GPS records of 14,692 taxis from January 2015. It was collected by GPS modules that were equipped in the taxies. The total number of GPS records is approximately 5.5 billion, which require storage of up to 100 G. The vehicle GPS records are described in Table 1. Each GPS record includes six fields: vehicle ID, time stamp, longitude, latitude, speed, and occupation status. 

## 4. Methodology

**Figure 1:**
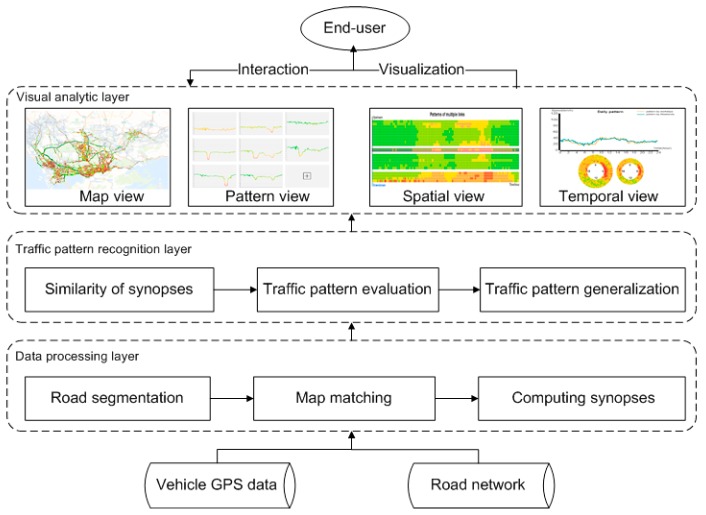
Workflow of the proposed framework.

We propose a three-layer framework for extracting and exploring traffic patterns, which is illustrated in Figure 1. In the first data processing layer, raw GPS records are processed to produce the synopses, which constitute a middle-tier data structure that represents traffic states of fine-grained road segments. In the second traffic pattern recognition layer, typical traffic patterns are recognized. The visual analytic layer provides interactions and intuitive visualization for the real-time exploration of traffic patterns. These layers are described in detail below.

### 4.1. Data Processing

Due to the dynamic nature of traffic, the traffic state varies over space and time. Instead of extracting traffic patterns from raw GPS records directly, we process raw GPS data into middle-tier synopses to accelerate the subsequent exploration. Formally, the synopsis is a traffic state description of a road segment during a period. The synopsis can be defined as a quadruples *M* = {*p*, *t*, *P, s*}, where *p* is the road segment, *t* is the time, *P* is the set of GPS records within the road segment *p*, and *s* is the corresponding traffic state. The length of the road segment and the time interval of the sequential synopsis are important as the fine-grained spatial and temporal units will significantly increase the required data storage. According to the first law in geography, “near things are more related than distant things” [55]. Traffic states on shorter road links are more homogeneous. Therefore, we segmented long road links into shorter segments. Similarly, a smaller time interval is also suggested. After intensive experimentation, we set the maximum length of a road segment to 200 m. If the length of a road link exceeds 200 m, we divide it to several segments. We set the time interval to 15 min, which is a typical value in transport studies and applications [56,57]. Regarding the set of GPS records, *P*, we filter the related records of a synopsis by the location of road segment *p* and the time *t*. Noted that taxis travel slowly or stop while looking for passengers, which will underestimate road traffic. Thus, we firstly filter out GPS records with the occupation status un-occupied. Raw GPS data are then related to the corresponding road segment by map-matching. As the used parallel map-matching method [7] is very fast, we can produce the synopsis on-the-fly when GPS records are streamed into the taxi monitoring system or with the post-processing mode. 

We regard the average travel speed as the traffic state. The value of the traffic state *s* can be computed using the velocity vector *s* = [$v_{1},\;v_{2},\;\ldots ,\;v_{n}$], as in Equation (1), where $v_{i}$ is the traffic speed from the GPS records, and *n* is the number of matched records. It is also essential to evaluate the reliability of the synopsis. Here, we present the variance of the speed to indicate the trustworthiness of the traffic state value, which is calculated via Equation (2). A large variance indicator suggests high uncertainty in the value of the traffic state. If no record is available, we set the value to “−1”, and the road link cannot be sensed during the corresponding 15 min using taxi GPS data.

(1) $$s=\frac{1}{n}\overset{n}{\underset{i=1}{\sum }}v_{i}$$

(2) $$S=\sqrt{\frac{1}{n-1}\overset{n}{\underset{i=1}{\sum }}{\left(v_{i}-s\right)}^{2}}$$

Finally, after computing all synopses, we can represent road traffic dynamic with a sequence of synopses. We stored all synopses in a geodatabase for the subsequent traffic pattern recognition, visualization, and exploration. Considering the imbalance of the GPS records, we created the spatial index and temporal index on synopses to accelerate the data query and retrieving. 

### 4.2. Pattern Recognition

We use time sequential synopsis to evaluate the dynamic variations of road traffic. Similarity measures have been widely used in pattern recognition, such as the Manhattan distance and the Pearson distance. Here, an algorithm, named "Trend and Value Distance (TVD)", is presented for evaluating the similarity of road traffic sequence. In contrast to many similarity measures that consider only the numeric values or the sequence order, the presented TVD algorithm integrates both to examine the similarity of two sequences. For a specified road and a time period from $t_{p}$ to $t_{q}$, let $SEQ_{\left\{p,\;t_{p},\;t_{q}\right\}}=\;\langle s_{\left\{p,\;t_{p}\right\}},\;s_{\left\{p,\;t_{p+1}\right\}},\;\ldots ,\;s_{\left\{p,\;t_{q}\right\}}\rangle$ be the sequential synopses. The *TVD* is described in detail in Algorithm 1. The numeric differences between two sequential synopses are firstly computed (Lines 6 to 13). The Pearson distance is calculated and used to examine the similarity of trends (Line 15). These are combined as the final TVD result (Line 16). The value of the TVD ranges from 0 to 1. The larger the TVD value, the higher the similarity of two synopses. Here, the TVD is used to evaluate the similarity of traffic dynamic of one road link between periods. It is also used to evaluate the similarity of the traffic dynamic of multiple road links.
**Algorithm 1** TVD**Input:** SEQ1, SEQ2: two time sequential synopses *μ*: adjusting parameter **Output:** TVD: distance value 1:  Length of sequential synopses: n = SEQ1.length 2:  Manhattan distance of SEQ1 and SEQ2: manhattan = 0 3:  Maximum Manhattan distance: max = 0 4:  Vector of the difference of SEQ1 and SEQ2: $\vec{diff}$ 5:  $\vec{e}$, where $e_{k}=1,\;k\in \left[1,\;2,\;\ldots ,\;n\right]$ 6:  **for**
i in n
**do** 7:   $abs_{i}=Math.abs\left(SEQ1_{i}-SEQ2_{i}\right)$ 8:   $manhattan+=\;abs_{i}$ 9:   $diff_{i}=SEQ1_{i}-SEQ2_{i}$ 10:   **if**
$abs_{i}>max$
**then** 11:    $\max=\;abs_{i}$ 12:   **end if** 13: **end for** 14: $DIS_{value}=1-\frac{manhattan}{n*max*\mu }$ 15: $DIS_{trend}=pearson\left(\vec{diff},\;\vec{e}\right)$ 16: $TVD=\;DIS_{value}*DIS_{trend}$ 17: **return**
TVD


For a road segment, if the similarity of two sequential synopses exceeds a threshold, it indicates a possible regular traffic pattern. We develop another algorithm, namely, “Traffic Regular Pattern (TRP)”, for identifying the hidden pattern, which is presented as Algorithm 2. There are three steps in TRP. Parameter *duration* determines the period of interest. The parameter *type* filters the days of the specified type during the period. In our system, there are three types to be used: *workday*, *weekend*, and *all* (workday and weekend). The first step (Lines 1–8) is to retrieve sequential synopses from the geodatabase according to the temporal query conditions that are specified by the end users. For example, the setting *duration* = “From 2015-01-01 to 2015-01-31” and *type* = “weekend” specifies that the synopses of all the weekends in January will be used to recognize a potential traffic pattern. The second step (Line 9) is to evaluate whether the traffic dynamics of a road in the specified period are a regular traffic pattern. We use DBSCAN [58] to cluster the sequential synopses. The TVD value is used to measure the similarity of two *sequences*. There are two parameters, namely, “eps” and “minpts”, in DBSCAN. The parameter “eps” is set according to experimentation. "Minpts" is not a constant; it is related to *sequences* that are filtered by parameters *duration* and *type*. The third step is to generalize a typical traffic pattern for each cluster from the sequential synopses (Lines 10–-21).
**Algorithm 2** TRP**Input:** allSynopses: All synopses duration: Specified temporal type eps: Parameter ‘eps‘ in DBSCAN
**Output**:
TRP: Collection of traffic patterns recognized 1:  Collection of daily traffic state sequences: collectionSequence=∅ 2:  All days during the period of allSynopses: days 3:  **for** each day in days **do** 4:    **if**
day within duration and day in type
**then** 5:      Push states of synopses on day into $\vec{sequence}$ chronologically 6:    **end if** 7:  **end for** 8:  Push every $\vec{sequence}$ into collectionSequence 9:  clusters=DBSCAN(collectionSequence, eps) 10:  **for** each $cluster_{i}$ in clusters
**do** 11:    sum=0 12:    Traffic pattern recognized of $cluster_{i}$: $TRP_{i}=\emptyset$ 13:    The number of sequences in $cluster_{i}$: $N=\;cluster_{i}.length$ 14:    **for**
k in N
**do** 15:      **for** each sequence in $cluster_{i}$
**do** 16:        $sum+=\;sequence_{k}$ 17:      **end for** 18:    **end for** 19:    $TRP_{i}\left[k\right]=\;sum/N$ 20:    Push $TRP_{i}\left[k\right]$ into TRP 21: **end for** 22: **return**
TRP


### 4.3. Interactive Traffic Pattern Explorative Analysis

**Figure 2:**
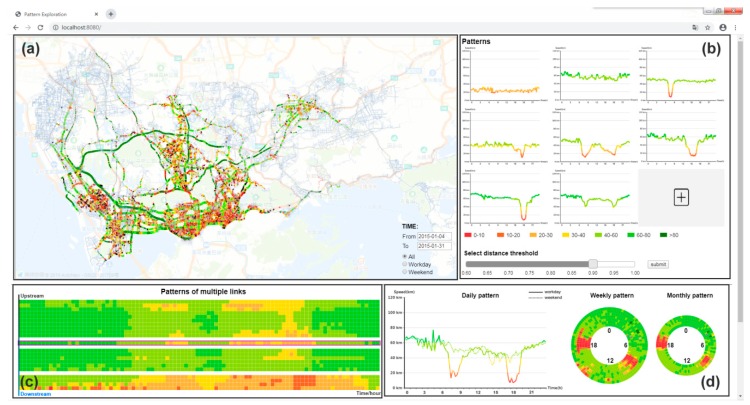
Interactive interface for exploring urban traffic pattern. (**a**) A map view that shows road links with the traffic state. (**b**) A pattern view for querying road links with similar traffic dynamics with a specified traffic pattern. By default, it gives the most typical road traffic patterns. (**c**) A spatial view that shows the traffic patterns of a selected road (with black frame in the middle) and its adjacent road links. (**d**) Temporal views that display the daily, weekly, and monthly traffic patterns in both a linear and radial diagram.

A web-based visual analytics system is developed for the real-time exploration of traffic patterns by end users. The interface is designed to comply with the classic visual exploration mantra, “Overview first, zoom and filter, details on demand” [59]. As illustrated in Figure 2, the designed interface consists of views (a, b) for global spatial and temporal pattern overview and views (c, d) for detailed exploration of roads of interest. 

For the overview navigation, a map view (a) is presented in the top left, with a digital map as a spatial reference to illustrate traffic state and traffic patterns by coloring the roads. Conventional map operations such as zooming and dragging are supported. End users can click or box select roads in the map view and set time conditions (i.e., *duration*, *type*, etc.) by the time components at the right-bottom of the map view to interactively explore traffic patterns of various roads according to their individual interests. Parameters used in the traffic pattern recognition layer are also set by end users. The extracted traffic patterns with high frequency are enumerated in the pattern view on the top-right (b) to show the typical cases of how the traffic state of a road segment changes over time. End users can click a pattern and observe its spatial distribution in the map view. The developed system also enables end users to add their patterns of interest as the target patterns by clicking on the roads.

Once traffic pattern of a specified road link has been interactively identified, it can be examined in detail from both the spatial and temporal perspectives. Spatially, the road traffic states of the adjacent 10 upstream links and 10 downstream links of the target link are displayed (c). Multiscale temporal dynamics of the road link are visually expressed by a line chart and radial charts (d). The line chart is able to better support numerical reading tasks, namely, users can easily perceive the speed at a time point. Radial charts are provided to support the cycle comparison tasks, e.g., over workdays or weekends. The radial layout is composed of several concentric rings, in which one ring displays one day traffic pattern in the clockwise direction. Examples with details will be presented in Section 5. 

## 5. Case Study

We conducted an experiment to evaluate the performance of the developed system. When raw GPS data streamed in, the synopses were computed and stored in the MongoDB database. The visual analytic system was implemented in JavaScript as a web-based system. We ran the experiment on a Dell desktop PC with a 3.40 GHz CPU and 8 G of memory. We reported several user cases to demonstrate the exploration of urban road traffic patterns using the developed interactive visualization system.

### 5.1. Regularity of Traffic States

One function of the developed system is to reveal the highly frequent traffic patterns of road links. The first case adopts the Visual Information Seeking Mantra "overview–zoom/filter–detail" [59] as the exploration strategy. For one road link, the TVD indicator was calculated to evaluate the similarity of the daily sequential traffic states in the workdays and the weekends. 

**Figure 3:**
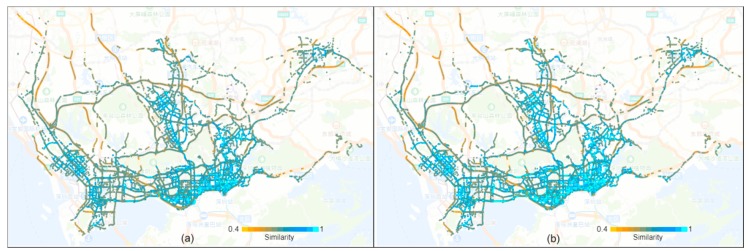
Similarity of the traffic patterns of each road link. (**a**) Workday. (**b**) Weekend.

Table 2 reports the descriptive statistics of road links with high self-similarity, which demonstrates the regularity of the road traffic dynamic. Figure 3 presents the spatial distribution of road links that differ in terms of self-similarity. The results demonstrate that 15,266 roads have a TVD indicator that exceeds 0.5, which cover 2294.2 km. These links cover most road links in the downtown areas, and main roads in the outer suburb. The TVD values were concentrated in the range of 0.8 to 0.9, with 9000 road segments in the workday and 8098 road segments in the weekend, respectively. In section of 0.9–1.0, the weekend mode has more road links, and according to the figure, it has more light-blue links. From the spatial perspective, many road links with high self-similarity are located in the urban center. A few roads with low self-similarity are located in the east Shenzhen and north Shenzhen. These results indicate the regularity of the road link traffic state. Therefore, it is possible to extract regular travel traffic patterns from massive vehicle GPS trajectories.

### 5.2. Temporal Dynamic of Road Traffic

**Figure 4:**
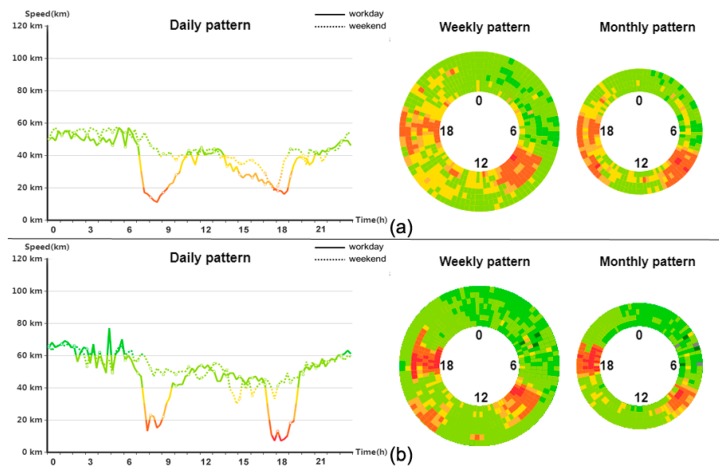
Temporal view of traffic patterns. (**a**) One road link. (**b**) Another road link located on the same road with opposite driving direction.

We zoom-in on the road links to investigate the temporal dynamics of traffic patterns. Upon clicking a link of interest on the map, its daily, weekly, and monthly traffic patterns are shown in the temporal view. Figure 4 shows an example of road links with typical traffic states. These two-road links are located on the same road but correspond to opposite driving directions. Due to large commuting volume, the road traffic significantly deteriorates during rush hour. The line chart in Figure 4a demonstrates that the travel speed decreases from 58 km/h to 18 km/h. The concentric circles are used to display weekly and monthly traffic patterns. One circle represents the traffic dynamic in one day. For the weekly pattern, the inner circle corresponds to Monday and the outer circle to Sunday. For the monthly pattern, the inner circle corresponds to the first week and the outer circle to the last week. The traffic pattern of the road in Figure 4a is stable on workdays and weekends. However, the monthly variation demonstrates that the road traffic deteriorates since more hours correspond to low travel speed week by week. Figure 4b suggests that the traffic dynamics differ between road directions. Both the weekly and monthly traffic patterns change minimally. The traffic patterns show high self-similarity on workdays and weekends from the weekly perspective, and stable traffic patterns are observed from the weekly perspective.

### 5.3. Traffic Pattern of Local Road Networks

**Figure 5:**
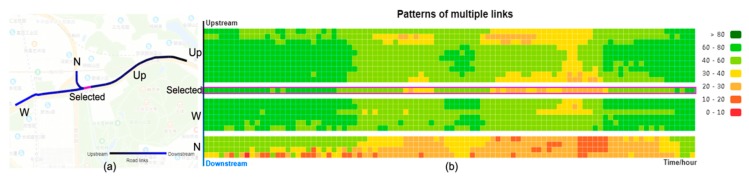
Traffic patterns of adjacent road links. (**a**) The topology of local road links. (**b**) The traffic dynamics.

We further examined the traffic pattern of local road networks. Upon clicking a road link, traffic patterns of its adjacent links from both upstream and downstream are visualized. Figure 5 shows an example and the associated daily traffic dynamic. At the selected road link, two roads (W and N) merge. The traffic patterns of adjacent road segments are similar on the long road, according to the traffic of the upstream and downstream roads. Furthermore, the topology influences the road traffic. Due to the merging of the travel flow at the selected roads, more vehicles gather at this intersection, thereby leading to changes in road traffic. According to the traffic of the downstream road in Figure 5b, traffic in these segments is worse than in the upstream segments. This friendly interactive tool enables us to examine the traffic dynamics of local road networks of interest. 

### 5.4. Traffic Pattern Exploration

**Figure 6:**
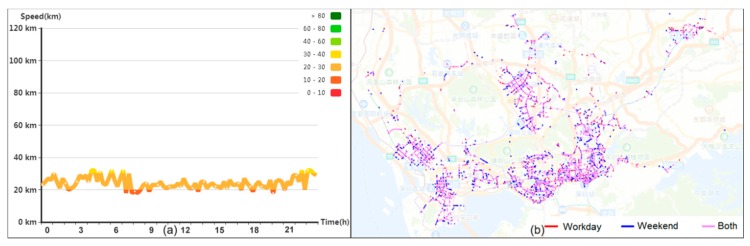
Most common pattern of all road links during a month. (**a**) The most common traffic pattern and (**b**) the road links with similar patterns.

The relationships of the traffic patterns among road links are also visualized. With the effective synopses, the proposed system supports efficient query of roads with similar patterns. Firstly, we answer the following question: “Which types of patterns are the most common?” We computed traffic pattern of each road link using one-month data and type “all”. Via the TRP Algorithm, the pattern of the largest cluster is identified, as shown in Figure 6a. This pattern, in which the speed fluctuates around 30 km/h all day, is the most common pattern. According to the spatial view in Figure 6b, most of the roads are branch roads in the urban center (Futian, Luohu, and Nanshan) and subcenter (Baoan, Longhua, and Longgang), where the travel speeds are limited by the transport law or travel flow. 

**Figure 7:**
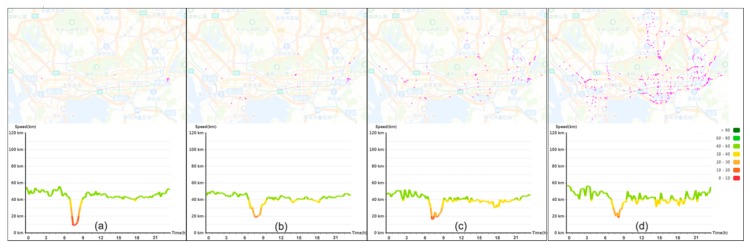
Results of road links with a specified pattern for different TV distance values. The similarity indicator is (**a**) 0.88; (**b**) 0.86; (**c**) 0.84; and (**d**) 0.82.

Facilitated by the pattern view, the question of “which roads share a similar pattern?” is further answered. Here, due to the importance of rush hour in the transport domain, we selected the morning rush hour for case study. We selected one road link with one typical morning rush hour traffic state as the target and queried roads with similar patterns. By specifying various similarity degrees, we obtained various sets of road links. Figure 7 shows the roads that were returned in response to input TV distance values, of 0.88, 0.86, 0.84, and 0.82. The spatial view displays the distribution of roads that have similar patterns. As the similarity degree is relaxed, increasingly, many roads appear. Meanwhile, the temporal change of the traffic states is amplified. From the line charts in the figure, the duration and the drop extent of the morning rush hour differ, although the deterioration of the traffic state in the morning rush hour is maintained.

**Figure 8:**
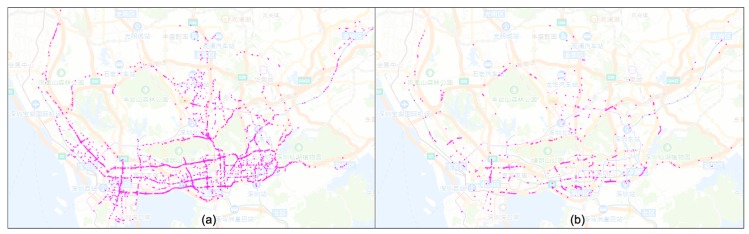
Spatial distributions of links during traffic rush hours. Similarity is (**a**) the morning rush hour and (**b**) the evening rush hour.

Next, we select patterns with “morning rush hour” and “evening rush hour” as the targets to explore roads that have similar patterns. Figure 8 displays the obtained results in the spatial view. According to Figure 8, many primary roads show the typical “morning rush hour” pattern, especially the main road in south Shenzhen. That is because many people who are living in north Shenzhen travel to work in the south. In contrast, the road with the evening traffic rush pattern is more spatially scattered. 

## 6. Discussion

### 6.1. Computing Performance

One of the key challenges in traffic pattern extraction is how to meet the need for real-time exploration leveraging accumulated big GPS data. Instead of processed massive GPS trajectories directly, the presented framework used the middle-tier synopses to extract traffic patterns for real-time exploration. The synopses can be computed on-the-fly when GPS records are streamed in or in a pre-processing stage. Therefore, the computation in the data processing layer does not affect the performance of the proposed interactive visual analytics system. In terms of storage, if the total length of the road network is *L* meters, the finest spatial scale unit is *m* meters, and the finest temporal scale unit is *n* minutes, then the number of synopsis in one day is computed as

(3) $$N_{daily}=\left(L/m\right)\times \left(24\times 60/n\right)$$

As *m* and *n* decreases, the number of synopses increases, and more storage space is needed. 

Table 3 presents an example of the storage space that is needed based on the road network data of Shenzhen City with various values of *m* and *n*. The raw GPS data size for this day is 3221 M. According to the table, if the spatiotemporal unit is "100 m and 5 min”, the storage space for the synopses is larger than that for the raw GPS data. Therefore, if the applications do not require the finest scale unit, the storage space will be reduced in most cases.

The number of synopses does affect the performance of real-time exploration in the traffic pattern recognition layer and the visual analytic layer. The finer the spatial and temporal units are selected, the bigger the number of synopses is computed. For the traffic pattern recognition layer, the computation performance depends on the size of synopses. Based on end users’ interactive setting including roads, *duration,* and *type* in TRP, synopses are retrieved from the geodatabase. The retrieving performance is related to the database used. Because the time complexity of the filtering is *O(1),* retrieving time on large volume of data will not be very long. Suppose *m* road links and *n* days are filtered by the specified parameters. Line 9 in the TRP calls the DBSCAN algorithm, which has a time complexity of O((m*n)^2^). The time complexity of Lines 10–21 is also O((m*n)^2^). In conclusion, the time complexity of the presented method is O((m*n)^2^).

In addition, both synopses computing in the data processing layer and in the traffic pattern recognition layer can be decomposed into independent subproblems, which can be solved by advanced distributed computing. Therefore, more computing cells will further improve the computing performance.

### 6.2. Scalability

The presented framework can be reused and extended. First, the framework is general to GPS data collected by many types of vehicles. In the proposed framework, raw GPS data are processed into synopses according to the location (including longitude and latitude) and the recording time. Traffic state is denoted by travel speed. Therefore, any GPS data that contains the fields “longitude, latitude, time stamp, and speed” can be accepted. GPS trajectories of various vehicles including bus, logistics vehicles, and private vehicles are potential data sources. According to official statistics, by the end of December 2018, the number of motor vehicles in Shenzhen was about 3.35 million (http://sztqb.sznews.com/PC/content/201812/10/content_525436.html). It shows the great potential of our framework for better urban traffic understanding. 

Second, the interactive visual analytics system can be extended for other transport applications. Due to the efficient middle-tier synopsis, the developed framework integrates the machines’ computing ability and the end users’ experience, supporting real-time interactive urban traffic exploration analysis. Although this study focuses on traffic, the developed framework can be easily extended to other traffic applications, i.e., reconstructing the evolution of traffic in multiple road intersections, revealing the traffic pattern under events, etc. Furthermore, combined with professional models [60], the developed system can also support innovative transport tasks. For example, by introducing the vehicle emission model, the developed system can be used in fine-grained transport carbon emission and air pollution analysis.

## 7. Conclusions

Understanding urban traffic patterns benefits traffic management and urban planning. This study proposes a three-layer interactive framework for the exploration of urban traffic patterns. Massive raw taxi GPS data are assigned to the synopses in the first data processing layer. The computed synopses are stored in geodatabase for quick retrieving to recognize traffic patterns in the traffic pattern recognition layer and the visual analytics layer. User-friendly interfaces are developed for the real-time exploration of complex urban traffic in a mega-city in the spirit of visual analytics. An experiment in Shenzhen was conducted using taxi GPS data that were collected over one month. Following end users’ interests, traffic patterns of both the specified area and the related period were computed and visualized in real-time. The proposed visualization interfaces show various traffic patterns in different forms intuitively, which facilitates comparison, perception, and understanding of citywide road traffic. The results demonstrate that the presented framework provides useful tools for road traffic analysis and future transport planning.

## Figures and Tables

**Table 1:** Description of the raw taxi GPS data.

Attribute	Description	Example
Vehicle ID	Unique identifier of the vehicle	117376
Time stamp	Recording time, with an accuracy of one second	2015-01-11 00:00:10
Longitude	Longitude when recorded	114.124967
Latitude	Latitude when recorded	22.610739
Speed	Instantaneous velocity when recorded	72
Occupation status	Whether passengers are in the taxi	1

**Table 2:** Descriptive statistics of road segments with repeated traffic states.

Table		0.5–0.6	0.6–0.7	0.7–0.8	0.8–0.9	0.9–1.0	Total
Workday	Number of road links	5	207	2753	9000	3301	15,266
	Total length of road links (km)	1.0	37.4	454.1	1335.8	465.9	2294.2
Weekend	Number of road links	9	273	2148	8098	4738	15,266
	Total length of road links (km)	1.7	48.8	351.6	1208.9	683.2	2294.2

**Table 3:** Required storage space of middle-tier data for various finest spatiotemporal scale unit for a dataset in 3221 M size.

Spatiotemporal Scale	5 min	15 min	30 min
100 m	3324 M	1108 M	552 M
200 m	1424 M	474 M	238 M
500 m	570 M	190 M	96 M

## References

[1] Chen C., Zhang D., Li N., Zhou Z.H. (2014). B-Planner: Planning Bidirectional Night Bus Routes Using Large-Scale Taxi GPS Traces. IEEE Trans. Intell. Transp. Syst..

[2] Li Z., Filev D.P., Kolmanovsky I., Atkins E., Lu J. (2017). A New Clustering Algorithm for Processing GPS-Based Road Anomaly Reports with a Mahalanobis Distance. IEEE Trans. Intell. Transp. Syst..

[3] Pang L.X., Chawla S., Liu W., Zheng Y. (2013). On detection of emerging anomalous traffic patterns using GPS data. Data Knowl. Eng..

[4] Mao Y., Zhong H., Xiao X., Li X. (2017). A Segment-Based Trajectory Similarity Measure in the Urban Transportation Systems. Sensors.

[5] Yang X., Stewart K., Tang L., Xie Z., Li Q. (2018). A Review of GPS Trajectories Classification Based on Transportation Mode. Sensors.

[6] Yang W., Ai T., Lu W. (2018). A Method for Extracting Road Boundary Information from Crowdsourcing Vehicle GPS Trajectories. Sensors.

[7] Xie J.Y., Tu W., Li Q., Chang X., Ma C.L., Li Z., Huang L. (2017). A parallel map-matching approach for large volume floating car stream data. Geomat. Inf. Sci. Wuhan Univ..

[8] Quek C., Pasquier M., Lim B.B.S. (2006). POP-TRAFFIC: A novel fuzzy neural approach to road traffic analysis and prediction. IEEE Trans. Intell. Transp. Syst..

[9] Wang S., Zhang X., Cao J., He L., Stenneth L., Yu P.S., Li Z., Huang Z. (2017). Computing Urban Traffic Congestions by Incorporating Sparse GPS Probe Data and Social Media Data. ACM Trans. Inf. Syst..

[10] Altintasi O., Tuydes-Yaman H., Tuncay K. (2017). Detection of urban traffic patterns from Floating Car Data (FCD). Transp. Res. Procedia.

[11] Scholz R.W., Lu Y. (2014). Detection of dynamic activity patterns at a collective level from large-volume trajectory data. Int. J. Geogr. Inf. Sci..

[12] Zhao J., Zhang F., Tu L., Xu C., Shen D., Tian C., Li X.Y., Li Z. (2017). Estimation of Passenger Route Choice Pattern Using Smart Card Data for Complex Metro Systems. IEEE Trans. Intell. Transp. Syst..

[13] Hou Z., Li X. (2016). Repeatability and Similarity of Freeway Traffic Flow and Long-Term Prediction under Big Data. IEEE Trans. Intell. Transp. Syst..

[14] Salamanis A., Margaritis G., Kehagias D.D., Matzoulas G., Tzovaras D. (2017). Identifying patterns under both normal and abnormal traffic conditions for short-term traffic prediction. Transp. Res. Procedia.

[15] Mahboubi Z., Kochenderfer M.J. (2017). Learning Traffic Patterns at Small Airports from Flight Tracks. IEEE Trans. Intell. Transp. Syst..

[16] Qiang X., Shuang-Shuang Y. (2018). Clustering Algorithm for Urban Taxi Carpooling Vehicle Based on Data Field Energy. J. Adv. Transp..

[17] Zou H., Yue Y., Li Q., Shi Y. A spatial analysis approach for describing spatial pattern of urban traffic state. Proceedings of the 13th International IEEE Conference on Intelligent Transportation Systems.

[18] Guo H., Wang Z., Yu B., Zhao H., Yuan X. TripVista: Triple Perspective Visual Trajectory Analytics and its application on microscopic traffic data at a road intersection. Proceedings of the 2011 IEEE Pacific Visualization Symposium.

[19] Naveh K.S., Kim J. (2019). Urban Trajectory Analytics: Day-of-Week Movement Pattern Mining Using Tensor Factorization. IEEE Trans. Intell. Transp. Syst..

[20] Oh S.D., Kim Y.J., Hong J.S. (2015). Urban Traffic Flow Prediction System Using a Multifactor Pattern Recognition Model. IEEE Trans. Intell. Transp. Syst..

[21] Xie Z., Lv W., Huang S., Lu Z., Du B., Huang R. (2019). Sequential Graph Neural Network for Urban Road Traffic Speed Prediction. IEEE Access.

[22] Shi C., Chen B.Y., Lam W.H.K., Li Q. (2017). Heterogeneous Data Fusion Method to Estimate Travel Time Distributions in Congested Road Networks. Sensors.

[23] Tu W., Li Q., Fang Z., Shaw S.L., Zhou B., Chang X. (2016). Optimizing the locations of electric taxi charging stations: A spatial—Temporal demand coverage approach. Transp. Res. Part C Emerg. Technol..

[24] Imawan A., Indikawati F.I., Kwon J., Rao P. (2016). Querying and Extracting Timeline Information from Road Traffic Sensor Data. Sensors.

[25] Terroso-Saenz F., Muñoz A., Cecilia J.M. (2019). QUADRIVEN: A Framework for Qualitative Taxi Demand Prediction Based on Time-Variant Online Social Network Data Analysis. Sensors.

[26] Kerner B.S., Demir C., Herrtwich R.G., Klenov S.L., Rehborn H., Aleksic M., Haug A. Traffic state detection with floating car data in road networks. Proceedings of the 2005 IEEE Intelligent Transportation Systems.

[27] Wilby M.R., Díaz J.J.V., Rodríguez Gonz’lez A.B., Sotelo M.A. (2014). Lightweight Occupancy Estimation on Freeways Using Extended Floating Car Data. J. Intell. Transp. Syst..

[28] Polson N., Sokolov V. (2018). Bayesian Particle Tracking of Traffic Flows. IEEE Trans. Intell. Transp. Syst..

[29] Yang S., Kalpakis K., Biem A. (2014). Detecting Road Traffic Events by Coupling Multiple Timeseries With a Nonparametric Bayesian Method. IEEE Trans. Intell. Transp. Syst..

[30] Sun J., Sun J. (2015). A dynamic Bayesian network model for real-time crash prediction using traffic speed conditions data. Transp. Res. Part C Emerg. Technol..

[31] Adu-Gyamfi Y., Sharma A., Knickerbocker S., Hawkins N., Jackson M. Reliability of Probe Speed Data for Detecting Congestion Trends. Proceedings of the 2015 IEEE 18th International Conference on Intelligent Transportation Systems.

[32] Tu W., Santi P., Zhao T., He X., Li Q., Dong L., Wallington T.J., Ratti C. (2019). Acceptability, energy consumption, and costs of electric vehicle for ride-hailing drivers in Beijing. Appl. Energy.

[33] Wang F., Hu L., Zhou D., Sun R., Hu J., Zhao K. (2015). Estimating online vacancies in real-time road traffic monitoring with traffic sensor data stream. Ad Hoc Netw..

[34] Li Q., Ge Q., Miao L., Qi M. (2012). Measuring Variability of Arterial Road Traffic Condition Using Archived Probe Data. J. Transp. Syst. Eng. Inf. Technol..

[35] Zhang Y., Haghani A., Zeng X. (2015). Component GARCH Models to Account for Seasonal Patterns and Uncertainties in Travel-Time Prediction. IEEE Trans. Intell. Transp. Syst..

[36] Daraghmi Y.A., Yi C.W., Chiang T.C. (2014). Negative Binomial Additive Models for Short-Term Traffic Flow Forecasting in Urban Areas. IEEE Trans. Intell. Transp. Syst..

[37] Chen M., Yu X., Liu Y. (2015). Mining moving patterns for predicting next location. Inf. Syst..

[38] Yue Y., Yeh G.O. (2008). Spatiotemporal traffic-flow dependency and short-term traffic forecasting. Environ. Plan. B Plan. Des..

[39] Cai P., Wang Y., Lu G., Chen P., Ding C., Sun J. (2016). A spatiotemporal correlative k-nearest neighbor model for short-term traffic multistep forecasting. Transp. Res. Part C Emerg. Technol..

[40] Chen W., Guo F., Wang F.Y. (2015). A Survey of Traffic Data Visualization. IEEE Trans. Intell. Transp. Syst..

[41] Lundblad P., Eurenius O., Heldring T. Interactive Visualization of Weather and Ship Data. Proceedings of the 13th International Conference Information Visualisation.

[42] Wood J., Dykes J., Slingsby A. (2010). Visualisation of Origins, Destinations and Flows with OD Maps. Cartogr. J..

[43] Andrienko G., Andrienko N., Dykes J., Fabrikant S.I., Wachowicz M. (2008). Geovisualization of Dynamics, Movement and Change: Key Issues and Developing Approaches in Visualization Research. Inf. Vis..

[44] Tominski C., Schumann H., Andrienko G., Andrienko N. (2012). Stacking-Based Visualization of Trajectory Attribute Data. IEEE Trans. Vis. Comput. Graph..

[45] Amini F., Rufiange S., Hossain Z., Ventura Q., Irani P., McGuffin M.J. (2015). The Impact of Interactivity on Comprehending 2D and 3D Visualizations of Movement Data. IEEE Trans. Vis. Comput. Graph..

[46] Sun G., Liang R., Qu H., Wu Y. (2017). Embedding Spatio-Temporal Information into Maps by Route-Zooming. IEEE Trans. Vis. Comput. Graph..

[47] Scheepens R., Willems N., Van de Wetering H., Andrienko G., Andrienko N., Van Wijk J.J. (2011). Composite Density Maps for Multivariate Trajectories. IEEE Trans. Vis. Comput. Graph..

[48] Andrienko G., Andrienko N., Hurter C., Rinzivillo S., Wrobel S. (2013). Scalable Analysis of Movement Data for Extracting and Exploring Significant Places. IEEE Trans. Vis. Comput. Graph..

[49] Guo D., Zhu X. (2014). Origin-Destination Flow Data Smoothing and Mapping. IEEE Trans. Vis. Comput. Graph..

[50] Wang Z., Lu M., Yuan X., Zhang J., Wetering H.v.d. (2013). Visual Traffic Jam Analysis Based on Trajectory Data. IEEE Trans. Vis. Comput. Graph..

[51] Andrienko G., Andrienko N., Bak P., Keim D., Wrobel S. (2013). Visual Analytics of Movement.

[52] Miranda F., Doraiswamy H., Lage M., Zhao K., Gonçalves B., Wilson L., Hsieh M., Silva C.T. (2017). Urban Pulse: Capturing the Rhythm of Cities. IEEE Trans. Vis. Comput. Graph..

[53] Lu M., Lai C., Ye T., Liang J., Yuan X. (2017). Visual Analysis of Multiple Route Choices based on General GPS Trajectories. IEEE Trans. Big Data.

[54] Liu H., Gao Y., Lu L., Liu S., Qu H., Ni L.M. Visual analysis of route diversity. Proceedings of the 2011 IEEE Conference on Visual Analytics Science and Technology (VAST).

[55] Tobler W.R. (1970). A Computer Movie Simulating Urban Growth in the Detroit Region. Econ. Geogr..

[56] Liu Z., Li Z., Li M., Xing W., Lu D. (2016). Mining Road Network Correlation for Traffic Estimation via Compressive Sensing. IEEE Trans. Intell. Transp. Syst..

[57] Yang B., Guo C., Jensen C.S. (2013). Travel cost inference from sparse, spatio temporally correlated time series using Markov models. Proc. VLDB Endow..

[58] Ester M., Kriegel H.P., Sander J., Xu X. A density-based algorithm for discovering clusters in large spatial databases with noise. Proceedings of the 2nd International Conference on Knowledge Discovery and Data Mining (KDD-96).

[59] Shneiderman B. The eyes have it: A task by data type taxonomy for information visualizations. Proceedings of the 1996 IEEE Symposium on Visual Languages.

[60] Zheng Y., Liu F., Hsieh H.P. U-Air: When Urban Air Quality Inference Meets Big Data. Proceedings of the 19th ACM SIGKDD International Conference on Knowledge Discovery and Data Mining ACM.

